# Modeling and Optimization of Ellagic Acid from Chebulae Fructus Using Response Surface Methodology Coupled with Artificial Neural Network

**DOI:** 10.3390/molecules29163953

**Published:** 2024-08-21

**Authors:** Junkai Wu, Fan Yang, Liyang Guo, Zunlai Sheng

**Affiliations:** 1 School of Pharmacy, Quanzhou Medical College, Quanzhou 362011, China; wujk1@hotmail.com; 2 College of Veterinary Medicine, Northeast Agricultural University, Harbin 150006, China; 3 Heilongjiang Key Laboratory for Animal Disease Control and Pharmaceutical Development, Northeast Agricultural University, Harbin 150006, China

**Keywords:** artificial neural networks, Chebulae Fructus, response surface methodology, ellagic acid

## Abstract

The dried ripe fruit of *Terminalia chebula* Retz. is a common Chinese materia medica, and ellagic acid (EA), isolated from the plant, is an important bioactive component for medicinal purposes. This study aimed to delineate the optimal extraction parameters for extracting the EA content from Chebulae Fructus (CF), focusing on the variables of ethanol concentration, extraction temperature, liquid–solid ratio, and extraction time. Utilizing a combination of the response surface methodology (RSM) and an artificial neural network (ANN), we systematically investigated these parameters to maximize the EA extraction efficiency. The extraction yields for EA obtained under the predicted optimal conditions validated the efficacy of both the RSM and ANN models. Analysis using the ANN-predicted data showed a higher coefficient of determination (*R*^2^) value of 0.9970 and a relative error of 0.79, compared to the RSM’s 2.85. The optimal conditions using the ANN are an ethanol concentration of 61.00%, an extraction temperature of 77 °C, a liquid–solid ratio of 26 mL g^−1^ and an extraction time of 103 min. These findings significantly enhance our understanding of the industrial-scale optimization process for EA extraction from CF.

## 1. Introduction

**Figure 1:**
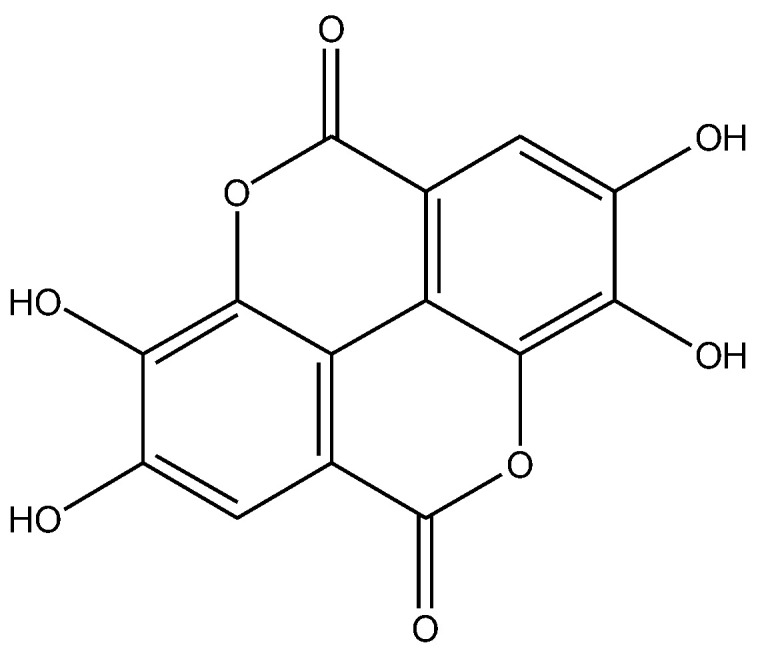
Chemical structure of ellagic acid (EA).

Chebulae Fructus (CF), known colloquially as “Hezi” within the annals of traditional Chinese medicine, comprises the dried, mature fruits of either *Terminalia chebula* Retz. or its variant, *Terminalia chebula* Retz. var. *tomentella* Kurt. This species, *T. chebula*, stands as a towering arboreal presence across South and Southeast Asia. In the Chinese Pharmacopoeia, CF is documented for its therapeutic applications, which encompass the treatment of chronic diarrhea and dysentery, hematochezia, rectocele, and respiratory ailments such as cough accompanied by dyspnea stemming from pulmonary insufficiency, as well as persistent coughing, sore throat and hoarseness [1]. Recent studies revealed a spectrum of chemical constituents within CF, predominantly featuring phenolic acids, tannins, and triterpenes, with the phenolic acids identified as the predominant bioactive substances [2,3]. Ellagic acid (EA, Figure 1), [C_14_H_6_O_8_, CASRN 476-66-4] as the principle active component of the polyphenol dilactones abundant in a diverse array of botanical sources such as berries, pomegranates, walnuts, cranberries, pecans, and various other plant-based foods, exhibits a multitude of pharmacological activities, including anti-inflammatory, anti-bacterial, antioxidant, anticancer, hypoglycemic, and hepatoprotective effects [4,5,6,7,8,9]. The presence and concentration of EA within CF are directly associated with the plant’s therapeutic effectiveness. As a predominant bioactive constituent, EA is fundamental to the medicinal attributes of the herb. The well-defined chemical characteristics of EA render it a prime candidate for the standardization and quality assurance of CF preparations, guaranteeing that these products meet the requisite standards for the active ingredient’s concentration. Moreover, the compound’s stability and bioavailability are pivotal in establishing its role as a pivotal quality marker for CF. The in vitro antitumor effects of EA against a spectrum of cancers, including nasopharynx [10], lung [11], colon [12], bladder [13], breast [14], and gastric [15] cancers, highlight its significance in CF and have spurred considerable research interest. The escalating number of pharmaceutical advancements has propelled research into enhancing EA yield, prompting the exploration of diverse advanced extraction techniques such as UAE (Ultrasonic-Assisted Extraction), MAE (Microwave-Assisted Extraction), and SWE (Supercritical Water Extraction). Nonetheless, traditional solvent extraction remains a widely employed technique for EA extraction, favored for its extensive applicability and cost-effectiveness. A high EA content is contingent upon a multitude of variables, e.g., the ethanol concentration, extraction time, extraction temperature, and solid–liquid ratio [16]. Consequently, optimizing the extraction yield of this targeted constituent is a crucial initial consideration in the process.

Recently, the impact of pivotal factors on phenolic acids, such as chebulinic acid, chebulagic acid, ellagic acid, etc., has been studied with orthogonal experimental designs by several investigators [17,18]. However, to the best of our knowledge, no study has been conducted on the optimization of the EA extraction parameters from CF through an integrated approach utilizing both the response surface methodology (RSM) and an artificial neural network (ANN). RSM is acknowledged as a robust experimental design method for multivariate regression analysis to maximize the desired responses. Nowadays, RSM has been extensively applied to improve and optimize procedures across the food, pharmaceutical, and chemical sectors [19]. Likewise, ANN, inspired by the intricate architecture of the human brain, serves as a versatile tool for data fitting, optimization, and predictive analytics [20]. ANN can make predictions by leveraging the relationships between the input and output variables, owing to its ability to discern and learn intricate functional relations. The conjunction of RSM and ANN has emerged as a potent strategy for enhancing and predicting the extraction efficiency of bioactive compounds from botanical sources [21,22,23]. This integrated approach highlights the synergistic benefits of RSM and ANN in accurately modeling complex extraction processes and determining the optimal conditions for extraction. Additionally, the Genetic Algorithm (GA), an efficient optimization technique for challenges pertaining to bioactive components, can be used to identify the optimal extremum within a given function [24]. The Backpropagation (BP) algorithm, proposed in 1986, is widely used for its precision in optimization, particularly in training artificial neural networks by adjusting the weights through the backpropagation of errors. The optimization outcomes can be obtained quickly by optimizing the process accurately with the BP algorithm after initial optimization utilizing GA. As a result, the application of ANN-GA modeling and optimization in various processes has been a subject of study in the literature [25,26]. Therefore, the integration of RSM and ANN for process optimization has garnered increased attention and interest over the past few years.

In this study, we set out to model and optimize the pivotal processing parameters (ethanol concentration, extraction temperature, liquid–solid ratio and extraction time) for the EA content extracted from CF by employing both RSM and ANN-GA models. Simultaneously, we undertook a comparative evaluation of RSM and ANN-GA modeling to ascertain their relative efficacy in maximizing the yield of the bioactive component.

## 2. Results and Discussion

### 2.1. Experimental Ranges from Screening Study

**Figure 2:**
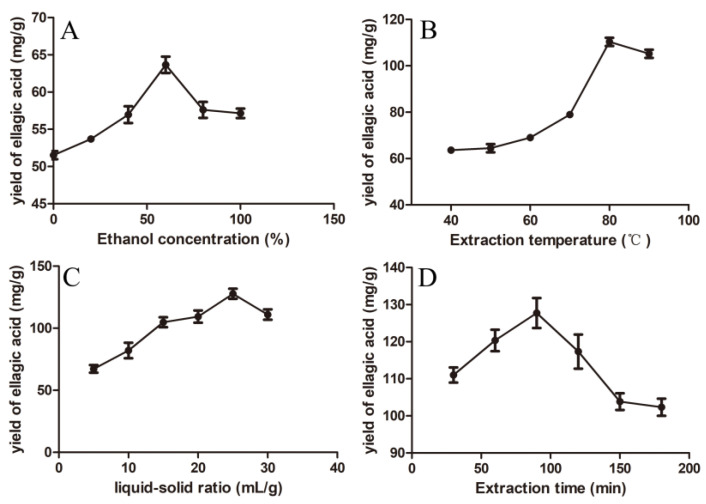
Effects of different factors on ellagic acid from CF. (**A**) Ethanol concentration, (**B**) extraction temperature, (**C**) liquid–solid ratio, and (**D**) extraction time.

In the single-factor experiments, the optimal ranges for the ethanol concentration, extraction temperature, liquid–solid ratio and extraction time were determined based on different extraction efficiencies for EA (Figure 2). The EA yield escalated with increasing ethanol concentration from 0% to 60%, achieving a peak with 60% ethanol solution (Figure 2A). As seen from Figure 2B, the extraction temperature that gave the highest EA content was 90 °C; however, considering that a 60% ethanol solution reaches its boiling point at this temperature, 100 °C was not deemed a viable extraction temperature. The EA yield reached the critical value at a liquid–solid ratio of 25 mL g^−1^, beyond which a slight decrease was observed (Figure 2C). Figure 2D illustrates a decline in the EA yield with extended extraction times from 90 min to 180 min, showing that 90 min is the central point for subsequent RSM experiments. The effective variable ranges derived from single-factor experiments were selected for the RSM using the Box–Behnken design (BBD), with ethanol concentrations ranging from 40% to 80%, extraction temperatures from 70 °C to 90 °C, liquid–solid ratios from 20 mL g^−1^ to 30 mL g^−1^, and extraction times from 60 min to 120 min. The choice of variable ranges for the extraction of various bioactive components can vary significantly, as factors such as the ethanol concentration, liquid–solid ratio, extraction temperature, and duration all play a crucial role in determining the extraction efficiency and facilitating mass transfer [27]. For instance, in the extraction of phenolic compounds from onion solid waste and *Inga edulis* leaves, researchers selected a quite different extraction time [28,29].

### 2.2. Response Surface Methodology Statistical Analysis and Model Fitting

According to the experimental design, the independent variables, along with the experimental and predicted values of EA, are presented in Table 1. The derived quadratic second-order equation, expressed in coded factors, that describes the EA yield is given in Equation (1).

Y = 121.52 + 1.80X_1_ − 2.98X_2_ + 13.01X_3_ + 6.78X_4_ + 1.09X_1_X_2_ + 0.2600X_1_X_3_ + 5.04X_1_X_4_ − 1.20X_2_X_3_ − 0.1150X_2_X_4_ + 16.47X_3_X_4_ − 25.23X_1_^2^ − 18.44X_2_^2^ − 23.29X_3_^2^ − 15.80X_4_^2^ (1)

where X_1_, X_2_, X_3_, X_4_ represent the ethanol concentration, extraction temperature, liquid–solid ratio and extraction time, respectively, while Y denotes the EA yield.

The statistical testing of the regression equation was ascertained via an *F*-test, and the analysis of the ANOVA results for the quadratic model is depicted in Table 2.

**Figure 3:**
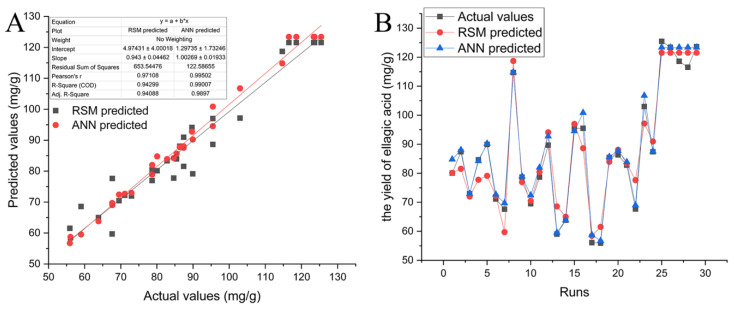
Relationship between the actual vs. predicted values using the RSM and ANN models (**A**); the matching between all the datasets (**B**).

The *F* value of 16.54 and *p* value < 0.0001 indicated that the model was highly significant for predicting the EA yield within the experimental framework. Additionally, the high *R*^2^ and adjusted *R*^2^ values of 0.9430 and 0.9409, respectively, confirm a well-fitted correlation between the experimental data and the quadratic model (Figure 3A). The normal plot of the residuals further validated the strong relationship between the predicted and actual data. The *p*-value for the lack of fit (0.0816 > 0.05) was nonsignificant in comparison to the pure error, further substantiating the model’s reliability. All of these indicated that the quadratic model was suitably equipped to evaluate the data derived from the RSM with a BBD [30].

### 2.3. Effects of Processing Parameters on Extraction

**Figure 4:**
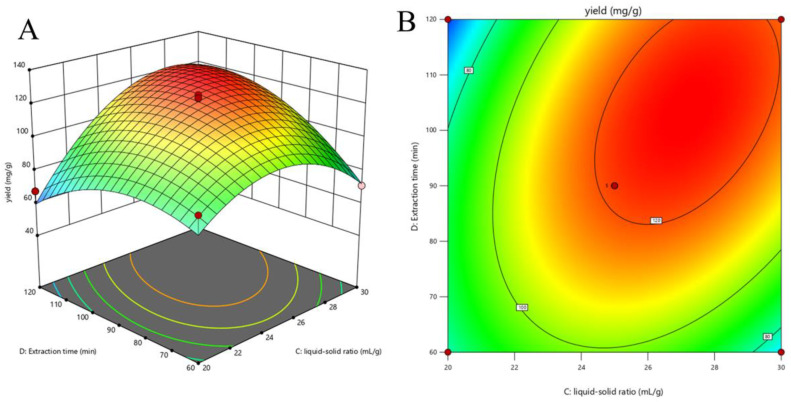
Response surface (3D, (**A**)) and contour plots (2D, (**B**)) showing extraction yield of ellagic acid (EA) as a function of the (**X_3_**) liquid–solid ratio and (**X_4_**) extraction time.

The ANOVA analysis (Table 2) revealed that the liquid–solid ratio exerts the most significant influence on the EA yield (*p* < 0.0001), while the effects of the ethanol concentration, extraction temperature and time are statistically insignificant (*p* > 0.0001). Considering the *F*-value of the independent variables, the order of the impact on the EA yield was liquid–solid ratio > extraction time > extraction temperature > ethanol concentration. The mutual interactions between the liquid–solid ratio (X_3_) and extraction time (X_4_) on the EA yield is visualized through the three-dimensional response surface and two-dimensional contour plots, as presented in Figure 4. Initially, the EA yield increased with the liquid–solid ratio, suggesting that a larger volume of solvent could dissolve more EA. However, a decline in the EA yield was observed when the liquid–solid ratio reached 30 mL g^−1^, potentially due to the decomposition of EA as a result of its strong antioxidant properties [31]. This finding contrasts with the optimal liquid–solid ratio reported by Wu et al. using ultrasonic-assisted extraction, which could be attributed to differences in the extraction methods used and material characteristics [32]. Furthermore, a study utilizing microwave-assisted extraction to isolate a bioactive pigment from chestnut shells achieved an ellagic acid content of 0.48 mg g^−1^ under the optimal conditions of an 800 W microwave power, a 12 min extraction time, and a solvent concentration of 0.115 mol l^−1^ NaOH [33]. These discrepancies emphasize the importance of considering the material properties, chemical composition, and other factors when selecting extraction methods for different medicinal materials. The optimal extraction conditions for achieving a maximum EA yield of 125.46 mg g^−1^, as predicted by the RSM, were determined to be an ethanol concentration of 60.00%, an extraction temperature of 78 °C, a liquid–solid ratio of 27 mL g^−1^, and an extraction time of 101 min.

Although some parameters are not statistically significant, they may still contribute to the overall explanatory power of the model. As indicated by Equation (1) and Table 2, the coefficients for parameters that are not significant are small, while those for significant parameters are large. We believe that retaining these parameters helps to capture the complexity and non-linear relationships in the experimental data. In practical applications, even parameters that are not statistically significant may hold practical importance for understanding and optimizing the process. Excluding non-significant parameters might lead to an overly simplified model, potentially missing important information. We have chosen to maintain a conservative approach to the model to avoid overfitting the data.

### 2.4. ANN Modeling and ANN Coupled with GA Optimization

**Figure 5:**
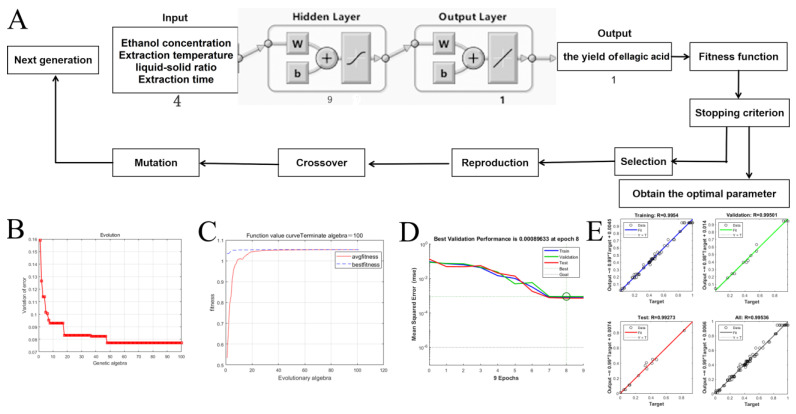
The optimal topology of the multilayer feed-forward neural network with the details of one neuron and the GA optimization steps, post-training (**A**); error variation of the ANN-GA model with different generation (**B**); ANN-GA model-simulated fitness function plot (**C**); the performance of the ANN model (**D**); regression plot showing the regression coefficient of the experimental data; and ANN model (**E**).

The ANN model can be constructed with a 4-9-1 topology within a multilayer perceptron framework. The mean squared errors (MSE) for the training, validation and testing datasets are depicted in Figure 5D. The training process was halted at epoch 8, at which point the MSE reached its minimum value. A correlation coefficient of determination (*R*^2^) for EA of 0.9970 demonstrated the high-level agreement between the experimentally obtained data and the predictions made by the ANN model. The results indicated that ANN modeling was a reliable predictor for the nonlinear data associated with EA extraction from CF, as detailed in Table 3. Subsequently, GA was applied to optimize the input space, with the aim of achieving the precise optimization of the EA extraction process. As illustrated in Figure 5C, the fitness metric grew progressively from the initial to the 48th generation, after which it plateaued within the range of generations 48 and 100. The optimal extraction conditions for a maximum EA yield of 125.46 mg g^−1^, as determined by the ANN-GA model, were as follows: ethanol concentration of 61.00%, extraction temperature of 77 °C, liquid–solid ratio of 26 mL g^−1^, and extraction time of 103 min.

### 2.5. Comparative Analysis of RSM and ANN

An optimized ANN-GA model was utilized to confirm the RSM results and further optimize the extraction variables for EA using an identical set of experimental data. The comparative evaluation of the RSM and ANN methods is typically predicated using the coefficient of determination (*R*^2^) and the relative error. The comparative analysis revealed that the ANN model outperforms RSM in terms of the predictive accuracy for the nonlinear data associated with EA extraction. The relative error values for the RSM and ANN models were 2.85 and 0.79, respectively, as presented in Table 3. It is indicated that when the experimental dataset employed in the RSM is afflicted with considerable error, the ANN methodology constitutes an advanced strategy for error reduction and the enhancement of precision. Analogous studies have suggested that the ANN method surpassed the RSM in predictive capability [34,35]. Consequently, the ANN-GA model can be effectively employed to provide accurate predictions of the EA content extracted from CF.

## 3. Materials and Methods

### 3.1. Materials and Chemicals

The fruits of *Terminalia chebula* Retz. (2.0 kg) were purchased from a local marketplace in Harbin, China, in October 2022. The samples were authenticated by professor Yanbing Li of the Heilongjiang University of Chinese Medicine, China. The ellagic acid standard (CAS 476-66-4) [36] was supplied by the National Institute for Food and Drug Control (Beijing, China). HPLC-grade acetonitrile was purchased from Kemiou Chemical Reagent (Tianjin, China). All reagents utilized in the extraction process, including ethanol and formic acid, were of analytical grade.

### 3.2. EA Extraction

The samples of pulverized CF (5 g) were subjected to reflux extraction under a designed ethanol concentration, solid–liquid ratio, extraction temperature and time according to the outcomes of preliminary single-factor experiments (Table 4). Immediately after the extraction procedure, the CF extract was promptly separated using centrifugation (3000 rpm, 5 min), and the residue was washed with eluent. Subsequently, the supernatant was filtered through a 0.45 μm millipore syringe filter and then stored in a dark place at 4 °C until further analysis.

### 3.3. EA Content Determination

The quantification of EA within the CF extract was ascertained using a validated rapid HPLC-UV method with minor modification [37]. The analytical process was conducted on a Shimadzu LC-20 chromatographic system (Shimadzu, Shenyang, China) equipped with a Dikma Inspire C18 column (100 × 4.6 mm I.D., 5µm, Dikma Technologies, Shenyang, China). The mobile phase was a mixture of water and formic acid (99.9/0.1 [*v*/*v*]), designated as solvent A, and acetonitrile was designated as solvent B, with a flow rate of 1.0 mL min^−1^. The gradient elution program was as follows: initial 0 min, 5% B; 25 min, 63.5% B; and 35 min, 63.5% B. The column oven was maintained at a consistent temperature of 30 °C, and each injection volume was 20 μL. The eluate was detected using a UV-vis detector set at a wavelength of 254 nm. The quantitative analysis of EA was performed by establishing a correlation between the concentration and peak area, with the EA content determined using the linear regression equations derived from the calibration curve.

### 3.4. Experimental Design and Statistical Analysis of RSM

Design-Expert 12.0.0 was used to ascertain the interaction among variables and to optimize the extraction conditions. Fischer’s *F*-test determined the second-order model equation at a probability (*p*) of 0.01 or 0.05. The adequacy of the model was determined by evaluating the lack of fit, the coefficient of determination (*R*^2^), and the *F*-test value obtained from the ANOVA that was generated. Preliminary single-factor experiments were conducted to establish the operational boundaries for the key process parameters: ethanol concentration (0–100%, *v*/*v*), extraction temperature (40–90 °C), liquid–solid ratio (5–30 mL g^−1^) and extraction time (30–180 min). Subsequently, the BBD strategy was used with four parameters and three levels [higher (+1), middle (0), and lower (−1)] for experimental design and data analysis (Table 4). A BBD matrix was constructed, comprising 29 independent experimental runs, to assess the impact on the EA content and predict the optimum extraction parameters (Table 1). Each experiment was triplicated, with the mean EA content serving as the experimental responses.

### 3.5. Artificial Neural Network Model with Genetic Algorithm

In the present study, the available experimental data from the RSM experiment were used for modeling the extraction of EA from CF using a multilayer perceptron-based feed-forward ANN in conjunction with GA optimization. The exact BP neural network architecture featured an input layer with four neurons (ethanol concentration, extraction temperature, liquid–solid ratio and extraction time), an output layer of one neuron (EA yield) and a hidden layer comprising nine neurons (Figure 5A). Traingdm was selected as the training parameter, and Mean Square Error (MSE) was utilized to assess the model’s precision. Several critical settings, predominantly the learning rate, momentum coefficient, number of epochs, and the configuration of hidden layers, were pivotal in influencing the neuronal simulation. The recommended momentum coefficient, learning rate, and hidden layer values of 0.7, 0.1 and 13, respectively, were used in this study to calculate the weights [38]. The GA optimization, integrated with the database and the ANN model trained via BP, delineated the optimal conditions yielding the maximum EA content. The average fitness values for each generation within the population were calculated using MATLAB R2019b software, with parameters set for the population size, maximum evolutionary algebra, crossover probability, mutation probability, and generation gap at 40, 100, 0.7, 0.1 and 0.95, respectively (Figure 5B,C). All computational analyses were performed using the Neural Network Toolbox of MATLAB version 9.10.0 (R2021a).

## 4. Conclusions

The present study aimed to choose a better method for optimizing the extraction process variables of EA from the ripe fruit of *T. chebula*. To achieve this, both the RSM and ANN-GA were utilized to investigate the effects of four pivotal parameters: ethanol concentration, extraction temperature, liquid–solid ratio, and extraction time. A comparative analysis based on *R*^2^ and the relative error revealed that the ANN model outperformed the RSM in terms of data fitting and predictive accuracy. The optimal extraction conditions derived from the ANN model were an ethanol concentration of 61.00%, an extraction temperature of 77 °C, a liquid–solid ratio of 26 mL g^−1^ and an extraction time of 103 min. Furthermore, the ANN model exhibited a more robust predictive capacity, with all *R* values exceeding 0.992, suggesting that the ANN may offer a more precise approach to EA extraction. Ultimately, the results of this research contribute to the enhancement of knowledge regarding the optimization of industrial EA extraction processes from CF. Additionally, this study demonstrates the potential of combining the ANN with the RSM or other models to further improve the precision and efficiency of the optimization process.

## Figures and Tables

**Table 1:** List of experimental values and predicted values from RSM and ANN.

Run	Independent Variables				The Yield of Ellagic Acid (mg g^−1^)		
	X_1_	X_2_	X_3_	X_4_	Actual Values	RSM Predicted	ANN Predicted
1	40	70	25	90	80.05	80.12	84.78
2	40	90	25	90	87.39	81.54	88.02
3	80	70	25	90	72.95	71.99	72.95
4	80	90	25	90	84.64	77.75	84.23
5	60	80	20	60	89.92	79.11	90.23
6	60	80	30	60	71.11	72.19	72.68
7	60	80	20	120	67.61	59.72	69.70
8	60	80	30	120	114.69	118.69	114.82
9	60	70	25	60	78.68	76.96	78.92
10	60	90	25	60	69.51	70.46	72.41
11	60	70	25	120	78.68	80.42	81.99
12	60	90	25	120	89.69	94.10	92.77
13	40	80	20	90	59.01	68.56	59.54
14	80	80	20	90	63.82	65.00	63.82
15	40	80	30	90	95.48	96.99	94.54
16	80	80	30	90	95.48	88.62	100.85
17	60	70	20	90	56.09	58.45	58.75
18	60	90	20	90	55.91	61.52	56.74
19	60	70	30	90	85.45	83.96	85.66
20	60	90	30	90	86.31	88.07	87.78
21	40	80	25	60	82.81	83.36	83.89
22	80	80	25	60	67.67	77.63	69.01
23	40	80	25	120	102.99	97.15	106.77
24	80	80	25	120	87.39	90.96	87.54
25	60	80	25	90	125.46	121.52	123.39
26	60	80	25	90	123.4	121.52	123.39
27	60	80	25	90	118.58	121.52	123.39
28	60	80	25	90	116.52	121.52	123.39
29	60	80	25	90	123.63	121.52	123.39

**Table 2:** ANOVA for quadratic model.

Source	Sum of Squares	df	Mean SQUARE	*F*-Value	*p*-Value	
Model	11,462.56	14	818.75	16.54	<0.0001	Significant
X_1_	38.7	1	38.7	0.7817	0.3916	
X_2_	106.68	1	106.68	2.15	0.1642	
X_3_	2032.16	1	2032.16	41.05	<0.0001	
X_4_	551.49	1	551.49	11.14	0.0049	
X_1_X_2_	4.73	1	4.73	0.0956	0.7618	
X_1_X_3_	0.2704	1	0.2704	0.0055	0.9421	
X_1_X_4_	101.81	1	101.81	2.06	0.1735	
X_2_X_3_	5.78	1	5.78	0.1168	0.7376	
X_2_X_4_	0.0529	1	0.0529	0.0011	0.9744	
X_3_X_4_	1085.37	1	1085.37	21.92	0.0004	
X_1_^2^	4128.66	1	4128.66	83.39	<0.0001	
X_2_^2^	2205.09	1	2205.09	44.54	<0.0001	
X_3_^2^	3517.75	1	3517.75	71.05	<0.0001	
X_4_^2^	1620.11	1	1620.11	32.72	<0.0001	
Residual	693.13	14	49.51			
Lack of Fit	635.98	10	63.6	4.45	0.0816	Not significant
Pure Error	57.15	4	14.29			
Cor Total	12,155.69	28				

**Table 3:** Predicted and experimental values of the responses at optimum conditions.

	Optimum Condition				Extraction Yield (mg g^−1^)		Relative Error
	X_1_	X_2_	X_3_	X_4_	Actual Value	Predicted Value	
RSM ANN	60 61	78 77	27 26	101 103	128.31 130.21	125.46 131	2.85 0.79

**Table 4:** Coding of experimental parameters and related levels.

Experimental Parameters	Unit	Symbols (X_i_)	Coded Values		
			Low (−1)	Medium (0)	High (+1)
Ethanol concentration	%	X_1_	40	60	80
Extraction temperature	°C	X^2^	70	80	90
liquid-solid ratio	mL g^−1^	X_3_	20	25	30
Extraction time	min	X_4_	60	90	120

## Data Availability

Data are contained within the article.
